# Pharmacology of boldine: summary of the field and update on recent advances

**DOI:** 10.3389/fphar.2024.1427147

**Published:** 2024-09-13

**Authors:** Juan C. Sáez, Justin C. Burrell, Catherine M. Cahill, D. Kacy Cullen, Lakshmi A. Devi, Ryan J. Gilbert, Zachary A. Graham, Vadim J. Gurvich, Leif A. Havton, Ravi Iyengar, Rajesh Khanna, Edmund F. Palermo, Mustafa Siddiq, Carlos A. Toro, Walter Vasquez, Wei Zhao, Christopher P. Cardozo

**Affiliations:** ^1^ Instituto de Neurociencias, Centro Interdisciplinario de Neurociencia, Universidad de Valparaíso, Valparaíso, Chile; ^2^ Center for Neurotrauma, Neurodegeneration and Restoration, CMC VA Medical Center, Philadelphia, PA, United States; ^3^ Department of Neurosurgery, Center for Brain Injury and Repair, Perelman School of Medicine, University of Pennsylvania, Philadelphia, PA, United States; ^4^ Psychiatry and Biobehavioral Sciences, University of California Los Angeles, Los Angeles, CA, United States; ^5^ Department of Pharmacology and System Therapeutics, Icahn School of Medicine at Mount Sinai, New York, NY, United States; ^6^ Department of Psychiatry, Icahn School of Medicine at Mount Sinai, New York, NY, United States; ^7^ Nash Family Department of Neuroscience, Icahn School of Medicine at Mount Sinai, New York, NY, United States; ^8^ Biomedical Engineering, Rensselaer Polytechnic Institute, Troy, NY, United States; ^9^ Albany Stratton VA Medical Center, New York, NY, United States; ^10^ Healthspan, Resilience and Performance, Florida Institute for Human and Machine Cognition, Gainesville, FL, United States; ^11^ Institute for Therapeutics Discovery and Development and Department of Medicinal Chemistry, College of Pharmacy, University of Minnesota, Minneapolis, MN, United States; ^12^ Department of Neurology, Icahn School of Medicine at Mount Sinai, New York, NY, United States; ^13^ Spinal Cord Damage Research Center, James J Peters VA Medical Center, New York, NY, United States; ^14^ Department of Pharmacological Sciences, Icahn School of Medicine at Mount Sinai, New York, NY, United States; ^15^ Institute for Systems Biomedicine, Icahn School of Medicine at Mount Sinai, New York, NY, United States; ^16^ Department of Pharmacology and Therapeutics, University of Florida, Gainesville, FL, United States; ^17^ Materials Science and Engineering, Rensselaer Polytechnic Institute, New York, NY, United States; ^18^ Department of Medicine, Icahn School of Medicine at Mount Sinai, Icahn School of Medicine at Mount Sinai, New York, NY, United States; ^19^ Department of Rehabilitation Medicine and Human Performance, Icahn School of Medicine at Mount Sinai, New York, NY, United States

**Keywords:** boldine, hemichannels, pharmacology, connexin (Cx), phramacokinetics

## Abstract

Over the past decade, boldine, a naturally occurring alkaloid found in several plant species including the Chilean Boldo tree, has garnered attention for its efficacy in rodent models of human disease. Some of the properties that have been attributed to boldine include antioxidant activities, neuroprotective and analgesic actions, hepatoprotective effects, anti-inflammatory actions, cardioprotective effects and anticancer potential. Compelling data now indicates that boldine blocks connexin (Cx) hemichannels (HCs) and that many if not all of its effects in rodent models of injury and disease are due to CxHC blockade. Here we provide an overview of boldine’s pharmacological properties, including its efficacy in rodent models of common human injuries and diseases, and of its absorption, distribution, pharmacokinetics, and metabolism.

## 1 Introduction

Natural compounds like boldine represent a vast reservoir of chemical diversity that can be explored for drug discovery and development. Investigating boldine’s mechanisms of action and potential therapeutic applications could lead to the development of new pharmaceuticals targeting conditions such as nervous system injuries and neurodegenerative disorders, liver disease, inflammation, infections, and cancer. Many traditional medicinal systems have used plants containing boldine for centuries to treat various ailments. If boldine or boldine-derived compounds prove effective in treating or preventing diseases, they could have a significant public health impact by reducing the burden of illness, improving patient outcomes, and potentially lowering healthcare costs associated with treating these conditions. Understanding the potential benefits of boldine also involves studying its safety profile, including potential side effects and interactions with other medications. Comprehensive research can help identify any risks associated with boldine use and inform healthcare professionals and consumers about its appropriate use. Here we summarize studies that provide insights regarding mechanisms by which boldine may produce its potential benefit in treating human diseases.

Pharmacological properties of the naturally occurring alkaloid boldine were first studied over 30 years ago when a series of papers demonstrated a potent ability of boldine to reduce oxidation of membrane lipids and proteins by reactive oxygen species (Speisky et al., 1991a; Cederbaum et al., 1992; Kringstein and Cederbaum, 1995). This anti-oxidant activity of boldine has been reviewed in depth elsewhere (O'Brien et al., 2006). The studies conducted in the last 20 years indicate that boldine may interact with specific receptors and ion channels, most notably by blocking movement of small molecules through connexin (Cx) hemichannels (HC). The current consensus is that boldine’s Cx HC blocking actions contribute much more to boldine’s therapeutic effects than its antioxidant activity. Research over the past 12 years has demonstrated that boldine reduces disease severity in various mouse and rat models simulating human diseases. This article aims to give a comprehensive summary of the journey of boldine’s discovery, its characterization, and its properties. It also intends to bring the reader up-to-date on the ongoing research into boldine’s medicinal benefits.

## 2 Boldine as an active ingredient in herbal remedies

Boldine is believed to be the major active ingredient present in the leaves and bark of the Boldo tree, which is indigenous to Chile (Speisky and Cassels, 1994). For more than a century, Boldo infusions, made by immersing Boldo leaves in hot water, have been utilized as a natural treatment for digestive disorders (Speisky and Cassels, 1994). Boldine was first identified in 1872 and was successfully synthesized soon thereafter (Bourgoin and Verne, 1872). The history of the study of its chemistry, and of analytical methods for quantification has been described elsewhere (Speisky and Cassels, 1994).

Whether boldine is the only biologically active molecule present in boldo is unclear. This uncertainty stems from several gaps in knowledge. While Boldo contains other alkaloids, as well as other organic moieties, much less is known regarding their biological and physiological activities. Another point of ambiguity arises due to the low water solubility of the boldine found in Boldo. This is elaborated further in the subsequent section. It brings up queries regarding the quantity of boldine that actually gets into the infusions when Boldo leaves are soaked in hot water. It is possible that other bioactive molecules are present in Boldo infusions. Because their bioactivities are not well studied, their properties are not reviewed in this article.

## 3 Physicochemical properties of boldine

**FIGURE 1:**
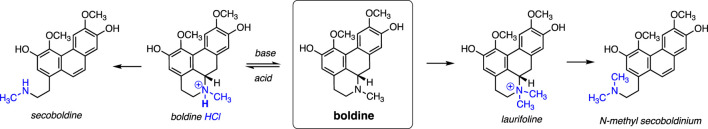
The chemical structure of Boldine and its semisynthetic derivatives.

Boldine (PubChem CID 10154, CAS #476-70-0) is an aporphine alkaloid substance with a molecular weight of 327.4 g/mol (Figure 1). Boldine is also known as uniboldina, boldin and (*S*)-boldine. The cumbersome IUPAC name, (*S*)-1,10-dimethoxy-6-methyl-5,6,6a,7-tetrahydro-4*H*-dibenzo [*de,g*] quinoline-2,9-diol, is rarely used. While originally identified in the leaves and bark of the Chilean Boldo plant (*Pemus boldus*), boldine has subsequently been identified in other plants including *Lindera umbellate* and *Damburneya salicifolia*. Boldine has a rigid polycyclic, aromatic structure that is hydrophobic and thus only sparingly water soluble, with a calculated water-octanol partition coefficient, logP ∼ +1.7 (O'Brien et al., 2006).

In terms of chemical reactivity, boldine displays two phenolic groups and one tertiary amine that are amenable to a wide range of possibilities for semi-synthetic derivatization (Namballa et al., 2022). The amine can be protonated under acidic conditions to yield the HCl salt form, which is somewhat soluble in citrate buffer of acidic pH (Akotkar et al., 2023). However, the pKa of the conjugate acid (the protonated tertiary amine group in boldine HCl) is predicted to be in the range of 9.7–10.8, based on pKa values for the related tertiary amines trimethyl- and triethylamine. A reliable literature value for the experimental pKa does not appear to be readily available. Assuming the predicted value for pKa, the compound will rapidly equilibrate to almost a completely neutral form in media of pH 7.4 (e.g., in serum), regardless of whether the HCl salt form or the neutral species is administered. Thus, the solubility limit in physiological conditions is presumably low. Hence, most boldine in serum is presumably protein-bound or may partition into the hydrophobic core of biomembranes.

Boldine is amenable to *N*-quaternization as a semi-synthetic modification that endows a stable cationic charge irrespective of pH, which greatly increases water solubility. This water-soluble form of boldine can be obtained by solubilizing boldine in hydrochloric acid then precipitating it with organic solvents (Urzúa, 1983).

The quaternized derivative of boldine is sometimes called “Laurifoline”, which in turn may further undergo ring-opening via β-elimination to yield the phenanthrene form, also called boldine methine or N-methyl secoboldinium, under conditions such as treatment with subcritical water (Borisenko et al., 2020). Boldine undergoes the same ring-opening process to yield the phenathrene product upon refluxing in aqueous ammonium acetate (Lee et al., 1995) This chemical modification does not abrogate its bioactivity, and may *enhance* antioxidant activity relative to native boldine.

## 4 Pharmacokinetics of boldine

### 4.1 Absorption, metabolism and elimination

Blood levels over time were measured in rats after oral doses of 25, 50 or 75 mg/kg in saline (Jimenez and Speisky, 2000). Peak blood levels were observed within 30 min after administration suggesting very rapid absorption (Jimenez and Speisky, 2000). Plots of log boldine concentration *versus* time revealed similar slopes for the decay in boldine levels across the three doses suggesting linear pharmacokinetics within the range of doses tested (Jimenez and Speisky, 2000). The elimination half-life was about 30–31 min (Jimenez and Speisky, 2000). Boldine was rapidly metabolized by suspensions of liver cells or in isolated perfused livers (Jimenez and Speisky, 2000).

Changes in boldine concentration after intravenous injection of either 10 or 20 mg/kg of boldine into rats via tail vein injection were also determined. Slopes for blood boldine concentrations over time were similar for the two doses and yielded a half-life of about 30 min, similar to that for oral administration (Jimenez and Speisky, 2000).

Tissue levels were determined for liver, brain and heart at 30 and 60 min after an oral dose of 50 or 75 mg/kg. Tissue levels increased with dose as expected. By far the highest levels were found in liver (72 nmol/g tissue at 50 mg/kg) but boldine was detectable in brain (18 nmol/g tissue at 30 min after 50 gm/kg) and heart (Jimenez and Speisky, 2000). The authors noted that tissue levels in liver (∼72 µM) were 4- to 5-fold greater than those needed to protect lipid membranes against peroxidation (Speisky et al., 1991a; Cederbaum et al., 1992).

A separate study evaluating oral and intravenous injection of boldine observed a shorter half-life of 12 min (Cermanova et al., 2016). Elimination of boldine included metabolism via glucouronidation and sulfation (Cermanova et al., 2016). In another study, the volume of distribution was estimated at 3.2 L in Lewis rats, a value much larger than the blood plasma volume, suggesting significant tissue uptake (Cermanova et al., 2016).

Bioavailability of boldine is likely to be less than 20%, most likely due to high first pass metabolism in the liver. In one study, peak blood levels were approximately 28 µM after an intravenous injection of 20 mg/kg but only 7 µM after oral administration of 25 mg/kg (Jimenez and Speisky, 2000). A second study found that the AUC for blood boldine levels was difficult to measure after oral administration but likely smaller than that for intravenous administration (Cermanova et al., 2016).

### 4.2 Tolerability and toxicology

The LD_50_ for intravenously administered boldine has been reported for mice as 450 mg/kg with death resulting from low blood pressure (Leboeuf et al., 1980). Lethal doses for oral administration of boldine were 1,000, and 1,250 mg/kg for guinea pigs and dogs, respectively (Speisky and Cassels, 1994). Lethal oral doses for boldine are thus approximately 20–100 fold or higher than the doses that are efficacious in animal models of disease (10–50 mg/kg) that are discussed in more detail below. Effects of boldine administered orally for 90 days at 50 mg/kg on liver and renal function and blood glucose were determined (Almeida et al., 2000). Importantly, long-term boldine administration did not increase aspartate amino transferase, alanine aminotransferase, cholesterol or bilirubin (Almeida et al., 2000). Serum glucose was modestly reduced by boldine, while creatinine and urea nitrogen levels were unchanged (Almeida et al., 2000). These studies confirm that at efficacious oral doses of 10–50 mg/kg, boldine does not have detectable toxicity to liver or kidney and may have beneficial effects on glucose uptake or metabolism.

### 4.3 Mutagenic properties of boldine

The potential of boldine to induce mutations has been evaluated in standard assays in *E. coli*, *Salmonella*, diploid *Saccharomyces cerevisiae* and haploid yeast (Moreno et al., 1991). These studies revealed no mutagenesis in prokaryotes and only rare recombination events in diploid eukaryotes. Boldine did not induce chromosome aberrations or chromatin exchanges in human peripheral blood lymphocytes at concentrations of up to 40 μg/mL; no effect of boldine was observed on mouse bone marrow cells when a single dose of boldine was administered by gavage at doses up to 900 mg/kg (Tavares and Takahashi, 1994). Effects on the fetus were evaluated by determining whether boldine caused fetal malformations or death in pregnant rats (Almeida et al., 2000). Oral administration of 500 mg/kg on days 1–5 and 7–12 led to no fetal deaths, with 1.53% of embryos lacking a tail. At 800 mg/kg/day at these same time points, 36% of embryo’s were resorbed, with malformations of the ear and paw occurring in about 3.5% of embryos. It should be noted that these studies used doses that were 10–50 fold greater than efficacious doses of 10–50 mg/kg/day used in the rodent studies described below.

## 5 Pharmacologic and physiologic effects of boldine

**FIGURE 2:**
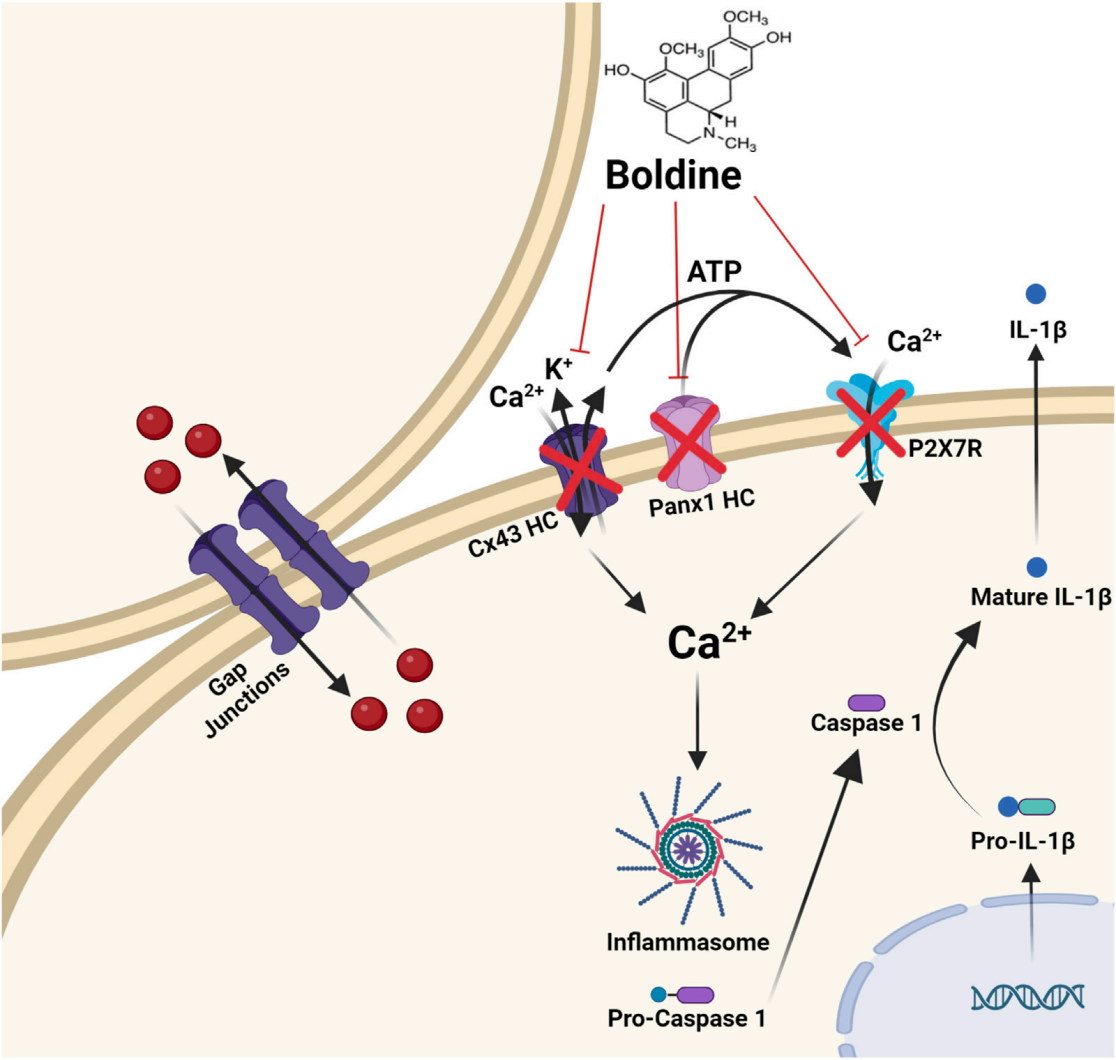
Molecular targets of boldine. Boldine inhibits connexin hemichannels (Cx HCs), pannexin 1 hemichannels (Panx1 HCs), and the purinergic receptor P2X7 (P2X7R). This channel blockade significantly reduces Ca2+ influx and K+ efflux, both of which are key activators of the inflammasome. As a result, boldine reduces the activation of caspase-1, leading to decreased production of IL-1β, a key outcome of inflammasome activation. Importantly, boldine does *not* inhibit gap junction channels formed by connexins.

Based upon literature searches using PubMed, boldine has been shown to interact with several cell surface proteins, including receptors and channels (Figure 2; Table 1). Below we have summarized findings from both *in vitro* and *ex-vivo* studies and commented on which of these activities appear to be observed *in vivo*.

**TABLE 1:** Potential targets of boldine.

Receptor	Pharmacologic effect of boldine	Concentrations or dose used	Citation
Connexin hemichannels	Blocked ethidium bromide uptake by rat mesangial cells incubated with high glucose and cytokines	100 µM	Hernandez-Salinas et al. (2013)
	Blocked ethidium bromide uptake into hippocampal slices, astrocytes and microglia at without altering gap junction communication	50 µM	(Yi et al., 2017)
	Reduced ethidium bromide uptake in HeLa cells expressing Cx26 or Cx30	50 µM	(Toro et al., 2023)
	Reduced ethidium bromide uptake in HC present in spinal cord activated by IL-1ß and TNF-α (mainly Cx43)	50 µM	(Toro et al., 2023)
	Blocked dye uptake by isolated myofibers from mice with endotoxemia (Cx43 and Cx45)	100 µM	(Cea et al., 2013)
Muscarinic Ach	Competitively antagonized acetylcholine-induced contraction of rat ilium	pA_2_ of 4.78	Speisky et al. (1991b)
Nicotinic ACh	Blocked Ach-induced twitch of denervated mouse diaphragm Reversed neuromuscular blockade by alpha-bungarotoxin	IC_50_ 13.5 µM 200 µM	Kang et al. (1998)
5-HT3A and 5-HT3-AB	Boldine was a competitive antagonist in Ca2+ influx, membrane potential and GF65630-binding assays	∼5 µM	Walstab et al. (2014)
Dopamine	Reduced *SCH 23390 and raclopride* binding in striatal slices Did not displace either ligand *in vivo* when administered at up to 40 mg/kg Did not alter apomorphine-induced behaviors (climbing, sniffing, grooming	0.4 µM for SCH 23390 0.5 M for raclopride 40 mg/kg	Asencio et al. (1999) (Asencio et al., 1999)
Farnasyl X	Boldine increased activity of a Farnysyl X reporter gene construct	Dose-response was U-shaped with a CMax at 5 µM	Cermanova et al. (2015)
P2X_7_R	Reduced Benzoyl-ATP induced Ca^2+^ entry	50 µM	Toro et al. (2023)
L-type Ca^2+^ channels	Reduced d-cis-diltiazem binding to rat cerebral membranes	IC10 33.28	Ivorra et al. (1993)

### 5.1 Membrane channel effects of boldine

Over the past several years, there has been increasing interest in effects of boldine on non-selective membrane channels such as Cx and pannexin1 HC as well as purinergic receptors. The first evidence that boldine blocked such membrane channels was the demonstration that boldine blocked dye uptake by a mesangial cell-derived cell line (MES-13 cells) (Hernandez-Salinas et al., 2013). These initial findings were followed by studies in cultured astrocytes and microglia (Yi et al., 2017), which primarily express Cx43. This experiment leveraged the previously documented ability of dyes such as ethidium bromide to pass through open CxHC. A follow-up experiment documented that boldine blocked dye-uptake into GFAP-positive cells via CxHC in hippocampal slices indicating reduced dye uptake by brain astrocytes (Yi et al., 2017). CxHC present on microglia were also inhibited in brain slices from mice (Yi et al., 2017). Because Cx43 is the most abundant Cx in the brain, it was assumed that much of the activity of boldine against dye uptake in cultured astrocytes and brain slices was attributable to blockade of this Cx. Intriguingly, pannexin 1 HC expressed in HeLa cells was also blocked by boldine (Yi et al., 2017). Subsequent experiments demonstrated that boldine reduced dye uptake by astrocytes present in spinal cord slices treated with TNF- α and IL-1ß to induce opening of HC (Toro et al., 2023).

Boldine blocked CxHC in freshly isolated skeletal myofibers from mice with endotoxemia (Cea et al., 2013). These finding were recapitulated by a muscle restricted double knockout of Cx43 and Cx45 (Cea et al., 2013). Unexpectedly, immunofluorescent staining of muscle from endotoxemic mice treated with boldine revealed that Cx43 was localized predominatly to the cytoplasm rather than the sarcolema. This finding suggests that in addition to blocking CxHC, boldine may alter trafficking of Cx through either greater internalization of sarcolemmal Cx or diversion from the ER or Golgi to cytoplamic compartments, the identify of which remains uncertain.

Moreover, boldine blocks CxHC *in vivo* and *in vitro* in skeletal myofibers of mice with dysfernilopathy (Cea et al., 2020). In the same work, using HeLa cells as an exogenous expression system, it was shown that a concentration of boldine that blocks Cx43 and Cx45 does not block Cx39 HC (Cea et al., 2013), suggesting some degree of selectivity as an inhibitor of CxHC.

More recently, direct effects of boldine on several membrane channels were tested in HeLa cell transfectants as these cells do not normally express Cx. These experiments demonstrated that boldine reduced dye uptake via Cx26 and Cx30 in the absence of other non-selective membrane channels permeable to small molecules (Toro et al., 2023).

Boldine also protects against endothelial dysfunction through a mechanism that has been proposed to result anti-oxidant actions (Lau et al., 2013a) or inhibition of an angiotensin II-mediated BMP4-oxidative stress cascade (Lau et al., 2013b). Interestingly, the antioxidant effect could be the result of the mechanism just described above; the angiotensin II-mediated oxidative stress cascade has been shown to be mediated by activation of Cx43 HC which are blocked by boldine (Gómez et al., 2018). In addition, inhibition of CxHC using peptides or antibodies reverses endothelial dysfunction induced by high glucose and proinflammatory cytokines (Sáez et al., 2018), supporting the interpretation that the protective effect of boldine in endothelial dysfunction could result from inhibition of CxHC.

The ability of boldine to reduce entry of Ca^2+^ via the P2X_7_R, an ion channel gated by extracellular ATP, was also evaluated. Boldine reduced benzoyl-ATP-induced Ca^2+^entry via P2X_7_R in a cell culture model (Toro et al., 2023). A similar result was recently reported in which HeLa cells expressing P2X_7_R-EGFP cultured in 22.5 mM glucose demonstrated increased Ca^2+^entry in the presence of 2 mM ATP that was blocked by 50 µM boldine (Cea et al., 2023).

Only a very limited investigation of the effects of boldine on other membrane channels has been reported. Specifically, Ivora and colleagues reported that boldine displaced an L-type Ca^2+^ channel ligand when added at moderately high concentrations (Ivorra et al., 1993). However, it is not known whether boldine blocks L-type Ca^2+^ channels. Boldine at very high concentrations (300 µM) induced muscle contraction, and potentiated effects of ryanodine (Kang and Cheng, 1998), an agonist for ryanodine receptors, which are large, calcium-gated calcium channels that release endoplasmic reticulum stores of calcium as part of excitation-contraction coupling. Boldine did not block the ATP-dependent transporter responsible for uptake of cytosolic calcium into the endoplasmic reticulum (Kang and Cheng, 1998). In sum, boldine can inhibit other non-selective channels and pore proteins on the cell membrane.

### 5.2 Adrenergic receptors

In studies of rat aortic strips, boldine was found to antagonize α-1-adrenergic receptor induced contraction elicited by norepinephrine (Ivorra et al., 1993). Moreover, boldine displaced the α-1-adrenergic receptor ligand prazocin from rat cortical membranes (Ivorra et al., 1993). In a follow-up paper from this same group, it was confirmed that S-(+) boldine blocked norepinephrine-induced contraction of aortic smooth muscle, suggesting that boldine is an α-1-adrenergic receptor antagonist (Chuliá et al., 1996). Physiological importance of this effect remains unclear. Hypotension has only been reported at a dose of 450 mg/kg, which is approximately 10-fold greater than oral doses used in studies of efficacy of boldine in rodent disease models. It remains to be studied whether boldine affects the functional state of other molecules involved in the contraction response of smooth muscles.

### 5.3 Acetylcholine receptors

Using rat ileum preparations, boldine was found to competitively antagonize acetylcholine-induced contractions (Speisky et al., 1991b), which are dependent on muscarinic acetylcholine receptors. Interpretation of these findings should take into account the fact that smooth muscle contraction also is influenced by Cx (Bol et al., 2017) and pannexin1 HC (Billaud et al., 2011; Sana-Ur-Rehman et al., 2017), which are blocked by boldine (Hernandez-Salinas et al., 2013; Yi et al., 2017). At higher concentrations, boldine has been shown to block acetylcholine-induced twitches of denervated mouse diaphragm and to reverse α-bungarotoxin induced neuromuscular blockade, indicative of nicotinic receptor blockade (Kang et al., 1998). Effects of boldine on neuromuscular function has not been evaluated *in vivo*. Most likely, tissue levels achieved with oral or i.v. dosing at 50 mg/kg are much lower than concentrations required to block acetylcholine receptors.

### 5.4 Dopaminergic receptors

Effects of boldine on dopamine receptor binding and apomorphine-induced behaviors have been reported. Apomorphine is an agonist at dopaminergic receptors, as well as serotonergic and α-adrenergic receptors (Ribarič, 2012). Studies in mice found no effect of boldine (40 mg/kg) on apomorphine-induced climbing, sniffing or grooming (Asencio et al., 1999). In rats, the highest intraperitoneal dose of boldine tested (40 mg/kg) reduced apomorphine-induced yawning and penile erection while lower doses had no effect (Asencio et al., 1999).

Effects of boldine on dopaminergic receptor binding have been tested. Experiments using rat brain slices demonstrated that boldine displaced two different dopamine receptor agonists with IC_50s_ in the low-micromolar range (Asencio et al., 1999). However, at a dose of 40 mg/kg i.p., boldine did not displace radiolabeled dopaminergic ligands *in vivo* (Asencio et al., 1999) although boldine is known to pass the blood-brain barrier (Jimenez and Speisky, 2000). Thus, *in vivo* anti-dopaminergic actions may only be observed at much higher doses than the efficacious outcomes discussed in later sections of this review.

Anti-nociceptive effects of boldine in mice were tested using a hot plate. Boldine prolonged the time to jumping in 60% of animals at 0.05–0.5 mg/kg s.c. and by 70% for 2–40 mg/kg s.c (Zetler, 1988).

### 5.5 Opioid receptors

Boldine has been reported to have weak antinociceptive effects in healthy mice that were not blocked by opioid receptor antagonists (Zetler, 1988). Hence, whether boldine binds to or activates opioid receptors is an open question and yet to be tested.

### 5.6 Serotonergic receptors

Potential effects of boldine on serotonin (5-HT) receptors were studied in transfected human embryonic kidney cells. Boldine displaced the 5-HT ligand GR65630 from both 5-HT3A and 5-HT3AB receptors and reduced 5-HT-induced calcium ion (Ca^2+^) entry with IC_50_ values of about 5 µM (Walstab et al., 2014). To date, there are no *in vivo* findings that expand on these results.

### 5.7 Farnysyl X receptor

The farnysl X receptor (FXR) is a heterodimeric nuclear receptor that plays critical roles in bile production and lipid metabolism (Chiang and Ferrell, 2020; Katafuchi and Makishima, 2022; Panzitt et al., 2022). The role of FXR in mediating boldine actions to stimulate bile flow (Cermanova et al., 2015) have been investigated using an FXR reporter gene construct. Boldine stimulated reporter gene activity with a U-shaped concentration response curve that demonstrated a maximal effect at 5 µM (Cermanova et al., 2015).

### 5.8 Other pharmacological and physiological actions of boldine

Boldine only weakly blocked contraction induced by KCl or elevated extracellular Ca^2+^ (Chuliá et al., 1996). Boldine did not antagonize histamine-induced contraction of tracheal smooth muscle (Chuliá et al., 1996). Boldine inhibited acetylcholinesterase with an IC_50_ of ∼8 µM (Mollataghi et al., 2012). Boldine reduced activity of phosphodiesterase IV with an IC_50_ 106 μM, while the IC_50_ of phosphodiesterase I-III was >300 µM (Ivorra et al., 1993).

## 6 Therapeutic potential of boldine in animal models of disease

There has been a growing interest in therapeutic potential of boldine over the past decade (Akotkar et al., 2023). Research in mouse and rat models of disease now support potential benefits of boldine in disorders of the nervous system, skeletal muscles, gastrointestinal system, bone and joint diseases and diabetes. Evidence of efficacy for treatment of disorders of these systems is summarized in Table 2 and discussed in more detail below. This listing includes all publications in which a functional endpoint was beneficially altered by boldine. As such, a small number of publications reporting *in vivo* effects of biochemical outcomes only are not discussed.

**TABLE 2:** Benefits of boldine in animal models of disease.

Disease model	Dose (mg/kg) or concentration/Route	Observed effects	Citation
*Nervous System*			
APPswe/PS1dE9 AD Mice	30 per day in water	Blocked astrocytic Ca^2+^ increase, ATP and glutamate release, mitochondrial ROS	Yi et al. (2017)
Spinal cord contusion Mice	50 per day orally	Boldine increased hindlimb function at every time point from 7 days on and reduced tissue loss	Toro et al. (2023)
Spinal cord transection Mice	50 per day orally	Boldine preserved muscle levels of glucose and amino acids and suppressed genes for protein ubiquitination/degradation	Potter et al. (2023)
Spinal cord transection Mice	50 per day orally	Boldine markedly altered lipid profiles in serum 7 days after transection and showed smaller changes in levels of circulating metabolites	Graham et al. (2023)
Middle cerebral artery occlusion Mice	8, 16 or 25 i.p	Smaller infarct, better memory function	de Lima et al. (2017)
Traumatic brain injury Mice	10, 20 or 30 i.p	In a weight drop model of TBI boldine reduced brain water and apoptosis in the cortex as well as slightly reducing cleaved caspase 3	Qiu et al. (2017)
Seizure Mice	1, 5, 10, 25 and 50 i.p	Boldine raised seizure threshold at 25–50 mg/kg	Moezi et al. (2019)
Muscle denervation and delayed nerve repair Rats	50 or 100 per day orally	Boldine reduced fiber atrophy after nerve transection for up to 4 weeks and elevated electrophysiologic response after nerve repair	Burrell et al. (2023)
*Skeletal Muscle*			
Dysferlin-deficient myogenic precursors and bLAJ mice (lacking dysferlin)	50 µM *in vitro*	Boldine reduced adipogenic differentiation of human myogenic progenitors and restored their ability to form myotubes Boldine improved Rotarod and 4-paw wire hanging performance	Cea et al. (2020)
STZ-induced diabetes Rats	50 per day by gavage	Boldine prevented myofiber atrophy (TA muscle) and reduced blood glucose 20%	Cea et al. (2023)
*Renal Disease*	*in vitro*		
Goldblatt 2K1C model Rats	50 per day by gavage	Reduced urinary proteinurea, histologic evidence of kidney damage and ACE-1 expression	Gómez and Velarde (2018)
Streptozotocin-induced diabetes Rats	50 per day by gavage	Boldine prevented hyperglycemia and blood pressure and urinary hyperproteinemia. In cultures of mesangial cells, boldine prevented hyperglycemia-induced increase in oxidative stress and CxHC activity	Hernandez-Salinas et al. (2013)
*Gastrointestinal Disease*			
Dextran-sulfate-induced colitis Mice	50 per day orally	Boldine reduced shortening of the colon	Pandurangan et al. (2016)
Acetic acid-induced colitis Rats	100 intrarectally 30 min prior to instilling acetic acid (single dose)	Boldine reduced edema, increased colonic fluid transport	Gotteland et al. (1997)
Ethanol-indomethacin-induced gastric erosions Mice	10, 100 or 1,000 orally	Boldine lowered gastric lesion area at 1 h after ethanol-HCl instillation into the stomach for the 10 and 100 mg/kg doses	Boeing et al. (2020)
Tert-butyl hydroperoxide damage Rats	200 µM *in vitro*	Using isolated rat hepatocytes, boldine reduced lipid peroxidation but did not increase cell survival	Bannach et al. (1996)
Acetaminophen-induced damage Mice	20 orally (single dose)	Boldine reduced serum AST and ALT as well as hepatic lipid peroxidation and expression of pro-inflammatory cytokines	Ezhilarasan and Raghunandhakumar (2021)
*Skeleton*			
Collagen-induced arthritis Mice	2 or 4 per day orally	Boldine reduced arthritis score	Zhao et al. (2017)
Ligature-induced periodontitis Mice	10, 20 or 40 per day orally	Boldine reduced bone resorption, osteoclast number	Cafferata et al. (2021)
*Cancer*			
HCT-116 and Saos-2 cell lines	5–80 μg/mL *in vitro*	Slowed cancer cell growth	Chandan et al. (2023)
T24 bladder cancer cells	Minimum effective concentration 200 µM *in vitro*	Reduced cell growth and cell viability	Gerhardt et al. (2014)
Human MDA-MB-231 and MDA-MB-468 breast cancer cells Rat LA7 mammary carcinoma Rats	IC 50–50 µM *in vitro* 50 or 100 i.p	Slowed cell proliferation Reduced tumor size	Paydar et al. (2014)
MCF-7 Bread cancer cells	5–80 μg/mL *in vitro*	Higher doses slowed tumor progression	Tomšík et al. (2016)

### 6.1 Nervous system disorders

A series of papers have reported diverse beneficial effects of boldine in models of diseases and injuries to the nervous system. Using a mouse APPswe/PS1dE9 model of Alzheimer’s disease (AD), boldine was shown to reduce several changes observed in cultured brain slices including blocking an increase in astrocyte cytosolic Ca^2+^, and release of ATP and glutamate (Yi et al., 2017). Using mitoSox, a mitochondrial-specific oxidative stress reporter, boldine reduced mitochondrial release of reactive oxygen species (ROS). Boldine reduced reticulon 3 immunostained dystrophic neurites, a finding interpreted as indicating reduced neuronal damage. While boldine did not appear to reduce glial reaction or amyloid accumulation, this paper established that boldine blocked astrocytic and microglial CxHC and the release of ATP and glutamate through them, thus reducing mitochondrial oxidative stress and neuronal damage. Cx have been implicated in other research investigating AD pathogenesis. Cx43 (GJA1) was identified as a key driver gene. Its expression was increased in post-mortem brain from AD patients (Kajiwara et al., 2018). Studies of cultured astrocytes expressing or lacking Cx43 revealed that the knockout altered many genes linked to AD, including some genes involved in metabolism of amyloid beta (Kajiwara et al., 2018). The Cx43 astrocytic knockout impaired amyloid beta phagocytosis (Kajiwara et al., 2018). Furthermore, boldine was shown to bind amyloid beta, to prevent amyloid beta-induced increases in cytosolic and endoplasmic reticulum levels of calcium ions (Toledo et al., 2021). While the specific roles of CxHC in the elevated levels of cytosolic calcium ions were not tested, results discussed elsewhere in this review suggest that amyloid beta could induce formation of HC on the cytoplasmic membrane of neurons.

The possible ability of boldine to reduce ischemia-related damage to the brain has been evaluated using a middle cerebral occlusion model in mice. Effects of administration of boldine 30 min before occlusion and daily for 5 days thereafter were evaluated (de Lima et al., 2017). Boldine improved aversive memory and novel object recognition at 25 mg/kg, associated with reduced infarct area and lower numbers of TNF-α and iNOS positive cells in the cortex and striatum.

The question of whether boldine would reduce indices of trauma in a mouse weight drop model of traumatic brain injury has been investigated. Boldine was administered 30 min after weight drop in these studies (Qiu et al., 2017). Indices of brain injury were determined 24 h after weight drop. Boldine reduced brain water content and levels of cleaved caspase 3 suggesting a protective effect. A behavioral test for grip strength was employed which demonstrated trends toward higher grip strength at early time points after brain trauma for boldine-treated groups although this difference was not statistically significant.

The impact of boldine on seizure threshold was assessed using a mouse model induced by pentylenetetrazole injection (Moezi et al., 2019). Boldine administration at doses of 25 or 50 mg/kg elevated seizure threshold and shortened the duration of myotonic contractions. This effect resembles that of vitamin C, suggesting a redox-based antiepileptic action. Another possibility is that both boldine and vitamin C decrease CxHC activity, albeit through different mechanisms: boldine may act as an inhibitor, while vitamin C could reverse the nitrosylation state of CxHC (Retamal et al., 2006; Retamal et al., 2007), reducing their activity, particularly in pathological conditions (Lillo et al., 2023). Recent evidence supports boldine’s antiepileptic action through inhibition of CxHC, as demonstrated by the significant reduction in neuroinflammation and physiological changes in temporal lobe epilepsy with the use of D4, a potent and selective CxHC blocker (Guo et al., 2022).

Effects of boldine on outcomes after spinal cord injuries (SCI) have also been evaluated. In one study, boldine was administered orally beginning 3 days after a moderate severity mid-thoracic spinal cord contusion in mice (Toro et al., 2023). Boldine-treated animals showed higher levels of function on the Basso mouse scale and horizonal ladder rung walk tests. Boldine increased spared white matter, a critical determinant of function after contusion SCI, and reduced immunofluorescence staining intensity for glial acidic fibrillary protein and Iba1. Confocal microscopy studies demonstrated that boldine also increased immunolabeling of proteins involved in axon growth and synaptic function.

A separate study evaluated effects of boldine on skeletal muscle following complete transection of the mid-thoracic spinal cord resulting in paralysis of hindlimb muscles (Potter et al., 2023). Again, boldine was administered orally at 50 mg/kg/day beginning 3 days after SCI. Effects of boldine on muscle were evaluated using transcriptomics, DNA methylomics, and metabolomics. Effects of boldine were most pronounced at 7 days after SCI. While boldine did not reduce muscle atrophy based on muscle wet weights, it did largely prevent alterations in skeletal muscle levels of glucose and several amino acids associated with prevention of the upregulation of gene expression involved in the ubiquitin-proteasome system, suggesting reduced protein turnover.

Effects of boldine on serum biomarkers at 7 days after SCI, the time at which the largest changes in muscle multiomic outcomes were observed, was investigated in a separate publication (Graham et al., 2023). In these studies, serum profiles of lipids and metabolites were evaluated using unbiased mass spectrometry techniques. Boldine mitigated SCI-induced changes in approximately 50 serum lipids including several phospho-inositols and ceramide. While boldine’s effects on levels of circulating metabolites were less robust, but some interesting trends were observed, including reduced serum serotonin.

Potential utility of boldine to mitigate the harmful effects of prolonged muscle denervation has been examined in a rodent model of delayed nerve repair. Effects of once daily oral boldine on magnitude of a compound muscle action potential and muscle fiber area of the denervated tibialis anterior muscle were investigated (Burrell et al., 2023). Boldine increased the evoked compound muscle action potential and reduced fiber atrophy up to 4 weeks after common peroneal nerve transection. Interestingly, boldine treatment decreased the intraneural Schwann cell expression of Cx43 in denervated nerves at 4 weeks post injury. In addition, boldine appeared to increase nerve electrophysiologic recovery and reinnervated muscle fiber size following delayed nerve repair. Collectively, these findings suggest boldine may prevent denervation-induced muscle atrophy after nerve injury, increasing the likelihood for functional recovery following delayed nerve repair. Addition work is necessary to elucidate potential direct axonal effects of boldine after nerve injury.

### 6.2 Skeletal muscle diseases and disorders

Emerging evidence supports the view that boldine reduces deleterious effects of at least some of the mutations that result in muscular dystrophy. In one study, a mix of cell culture and *in vivo* studies was performed (Cea et al., 2020). Using cells from human dysferilinopathy patients, boldine was found to switch the cell fate toward myogenic differentiation rather than adipogenic differentiation and to increase fusion potential of these precursor cells. This action of boldine was linked to lower expression of the adipogenic differentiation factor PPARγ. In bLAJ mice lacking dysferlin, 8-weeks of boldine reduced fatty accumulation in muscle, lowered PPARγ, increased performance on RotaRod and 4-paw wire hanging tests and reduced serum creatine kinase activity.

A separate study screened for the ability of boldine as a drug candidate for myotonic dystrophy (Álvarez-Abril et al., 2023). Using a Drosophilla reporter gene system, boldine lowered activity of the reporter and the number of ribonuclear foci, two indicators of myotonic dystrophy pathogenesis in this system. Boldine also reduced numbers of ribonuclear foci in cell lines derived from myotonic dystrophy patients. In further studies using a mouse model, boldine reduced ribonuclear foci, splicing modulation of the sarcoplasmic reticulum Ca^2+^ ATPase 1 (*Serca*) or the chloride channel (*Clcn1*), and myotonia when injected intramuscularly but not upon systemic administration.

The ability of boldine to reduce myofiber atrophy in diabetes has also been tested. Myofiber atrophy measured at 5 weeks after streptozotocin injection was significantly reduced by treatment with boldine (Cea et al., 2023) associated with a slight reduction in plasma glucose, possibly due to an incomplete CxHC inhibition. Culture of primary myofibers in 8 mM glucose increased ethidium bromide uptake; this increased membrane permeability was blocked by the CxHC blocker lanthanum ion and was prevented by boldine (Cea et al., 2023).

### 6.3 Renal diseases

The ability of boldine to slow kidney disease has been evaluated in rodent models of hypertension and diabetes. In a rat model of hypertension caused by placing a clip around one renal artery, administration of boldine once daily by gavage improved creatinine clearance and reduced proteinuria while reducing chronic changes in the kidney including deposition of collagen (Gómez and Velarde, 2018). In addition, boldine blocked hypertension-induced increased angiotensin converting enzyme-1 expression and increased TGF-ß expression (Gómez and Velarde, 2018).

Effects of boldine on the progression of diabetic kidney disease have also been tested in a rat model of streptozotocin-induced diabetes. Boldine prevented proteinuria and reduced histologic evidence of glomerulosclerosis (Hernandez-Salinas et al., 2013). Of interest, boldine was found to block ethidium bromide entry into cultured mesangial cells when these cells were incubated under high glucose conditions in the presence of TNF-α and IL-1ß indicating reduced movement of this dye through CxHC (Hernandez-Salinas et al., 2013).

### 6.4 Gastrointestinal diseases

Boldine was found to mitigate changes to the gastrointestinal system in several animal models. In one, boldine attenuated gross anatomical changes of the colon induced by dextran-sulfate ingestion (Pandurangan et al., 2016). In another study, pre-treatment with boldine reduced tissue edema and loss of colonic fluid transport resulting from acetic acid (Gotteland et al., 1997). Boldine also reduced damage to the stomach lining caused by ethanol and indomethacin (Boeing et al., 2020).

### 6.5 Hepatoprotective effects

Early studies reported hepatoprotective effects of boldine in several experimental systems. In an animal model of hepatotoxicity caused by acetaminophen overdose, boldine reduced the elevation of blood levels of liver enzymes and reduced lipid peroxidation in liver (Ezhilarasan and Raghunandhakumar, 2021). Consistent with these findings, boldine reduced lipid peroxidation caused by tert-butyl hydroperoxide in primary hepatocyte cultures (Bannach et al., 1996). In addition, boldine, has demonstrated hepatoprotective effects in rat models of non-alcoholic fatty liver disease (Zagorova et al., 2015), cholestasis and cirrhosis (Heidari et al., 2017), and diethylnitrosamine‐induced hepatocarcinogenesis (Subramaniam et al., 2019).

While use of boldo (from which boldine is isolated) is generally regarded as safe (Speisky and Cassels, 1994), there have been at least three case reports suggesting that hepatotoxicity might result from boldo consumption (Piscaglia et al., 2005; Ribeiro et al., 2017; Oliveira Sa et al., 2020). Given that boldo contains numerous alkaloids, the relevance of these case reports to the safety of boldine remains unclear. For example, boldo essential oil contains a toxic compound known as ascaridiol; boldo essential oil is therefore contraindicated for individuals with gallbladder stones or liver issues. Hepatotoxicity of long-term administration of boldine at doses found to be efficacious in rats (Table 2) did not appear to have hepatotoxic effects.

### 6.6 Diseases of the joints and skeleton

The literature suggests protective effects of boldine on bones and joints. An evaluation of effects of boldine on changes induced by intra-articular injection of collagen found that boldine reduced arthritis scores in this mouse model of inflammatory arthritis (Zhao et al., 2017). This protective effect was attributed to suppression of osteoclast formation based on a network pharmacology analysis (Zhao et al., 2017), a result that needs to be confirmed experimentally.

Effects of boldine on erosion of bone of the mandible was evaluated using a ligature-induced periodontitis model in mice. In these studies, boldine reduced bone resorption and osteoclast number (Cafferata et al., 2021).

### 6.7 Possible common mechanisms of boldine across disease models

It is noteworthy that many effects of conditional CX knockouts or CxHC blockers such as peptide 5 are phenocopied by boldine. For example, the ability of boldine to reduce myofiber atrophy of the tibialis anterior muscle after common peroneal nerve transection (Burrell et al., 2023) parallels the protection against denervation atrophy of fast muscle fibers resulting from a conditional knockout of Cx43 and Cx45 in skeletal muscle (Cea et al., 2013). Similarly, both the double knockout and boldine attenuated diabetes-induced myofiber atrophy (Cea et al., 2023). In another example related to skeletal muscle, physical performance of mice lacking dysferlin was improved by conditional knockouts of Cx43 and Cx45 and by boldine (Fernández et al., 2020). A common feature of these muscle disorders is increased sarcolemmal CxHC expression and increased membrane permeability to ethidium bromide and Ca^2+^ (Cea et al., 2013; Fernández et al., 2020; Cisterna et al., 2020). Other examples also exist. Peptide 5, inhibitory monoclonal antibodies against Cx43, boldine and conditional knockouts of Cx30 and Cx43 in astrocytes all reduced tissue injury and improved functional outcomes after spinal cord contusion (Toro et al., 2023; O'Carroll et al., 2013; Huang et al., 2012; Zhang et al., 2021).

Common mechanisms downstream of open CxHC may also be regulated as a consequence of blocking passage of Ca^2+^, ATP, glutamate and other small molecules through open CxHC by boldine. Cea and colleagues suggested that activated sarcolemmal HC trigger the inflammasome, leading to the expression and release of IL-1ß and TNF-α (Cea et al., 2013). Elevated cytosolic Ca^2+^ levels have been observed in various skeletal muscle disease models, with genetic deletion of Cx43 and Cx45 preventing these increases (Cea et al., 2013; Fernández et al., 2020; Cisterna et al., 2020). Boldine has been shown to decrease the expression of pro-inflammatory cytokines in various disease models, such as SCI (Toro et al., 2023) and acetaminophen-induced liver damage (Ezhilarasan and Raghunandhakumar, 2021).

It has been shown that boldine reduces the circulating levels of glucose and part of effect might result from Its inhibitory action starting at concentrations below 50 μM on at least three enzymes of the gluconeogenic pathway: phosphoenolpyruvate carboxykinase, fructose-bisphosphatase-1, and glucose 6-phosphatase. Conversely, boldine also increased glycolysis from glycogen-derived glucosyl units. Therefore, it has been proposed that the direct inhibition of hepatic gluconeogenesis by boldine, combined with the increase of glycolysis, could be an important event behind the diminished hyperglycemia observed in boldine-treated diabetic rats (Silva et al., 2023). Nevertheless, it remains to be demonstrated that inhibition of gluconeogenic enzymes and activation of glycolysis reproduce the boldine-induced outcome. On the other hand, it was recently demonstrated that skeletal myofibers of diabetic mice express Cx43 and Cx45 and form functional HC in the sarcolemma (Cea et al., 2023). Interestingly, treatment with boldine reduced the activity of these HC *in vivo* and *in vitro*, and reduced the glycemia as well (Cea et al., 2023). Since skeletal muscle comprise about 40% of the body mass of a young adult, it is logical to propose that by recovering the optimal functional state of the skeletal muscle, more glucose could be taken up from the circulation and consumed such that glycemia would be drastically reduced. In agreement with the effect of boldine on CxHC, it was demonstrated that diabetic mice deficient in Cx43 and Cx45 expression in myofibers also demonstrate a drastic reduction in glycemia (Cea et al., 2023).

The signal that triggers the opening of CxHC may vary depending on the disease model, potentially involving factors such as hyperglycemia (Hernandez-Salinas et al., 2013), FGF-1 (Garré et al., 2010), and IL-1ß combined with TNF-α (Toro et al., 2023). Once CxHC are opened, a feed-forward loop is initiated, potentially amplifying the original signal through various parallel mechanisms. ATP release could activate P2X_7_R or other purinergic receptors, enhancing the initial inward Ca^2+^ current. Elevated cytosolic Ca^2+^ levels may trigger increased mitochondrial respiration, release of signaling lipids, and activation of NOS, leading to oxidative stress. This oxidative stress, combined with high cytosolic Ca^2+^, could activate the inflammasome, increase transcription and release of pro-inflammatory mediators, thus perpetuating the initial signal. Notably, both boldine (Toro et al., 2023; Pandurangan et al., 2016) and conditional Cx knockouts (Cea et al., 2013) have been shown to reduce NF-kB signaling, providing evidence for this model. It is important to consider that the initial signal may be either extrinsic or intrinsic. For instance, denervation-induced atrophy exemplifies an intrinsic signal, where the loss of low-level tonic acetylcholine release from the motor neuron triggers *de novo* expression of CxHC, increased membrane permeability, activation of NF-kB, and elevated cytokine expression (Cisterna et al., 2020). In tissue trauma models such as SCI, circulating immune cells gain access to tissues because of damage to the blood brain barrier, while resident immune cells (e.g., microglia) are activated by tissue debris, resulting in local release of IL-1ß and TNF-α. While much of this model remains to be tested, it provides a conceptual, unifying framework for interpreting the growing number of studies of roles of CxHC in disease and of the mechanism and efficacy of boldine for treating medical conditions.

## 7 Concluding remarks

Boldine, an orally active alkaloid found in nature that has demonstrated good tolerability in rodents at doses up to 100 mg/kg when administered orally or parenterally. Its efficacy has been shown across various animal models of disease or injury to the nervous system, skeletal muscle, bone, gastrointestinal system, and kidney. In every case tested, open CxHC have been discovered in the cytoplasmic membranes of cells in the diseased tissue or organ, and conditional knockouts of the Cx expressed in these tissues recapitulates the effects of boldine. While the mechanisms by which CxHC appear in the cytoplasmic membrane vary, there are universal consequences of HC that include increased cytosolic calcium and inflammasome activation, together with release of ATP. Boldine blocks this cascade of deleterious signals, blunting or blocking expression and release of pro-inflammatory mediators which provide a feed-forward signal to amplify and perpetuate the deleterious changes induced by initial opening of CxHC. Accumulation of extracellular ATP may further amplify this initial signal by binding to purinergic receptors such as P2X_7_ receptor thereby increasing the inward flow of calcium ions. Boldine is thus an attractive and exciting candidate for continued investigation as a therapeutic agent.

## References

[B1] Akotkar L. Aswar U. Ganeshpurkar A. Raj R. Pawar A. (2023). An overview of chemistry, kinetics, toxicity and therapeutic potential of boldine in neurological disorders. Neurochem. Res. 48, 3283–3295. 10.1007/s11064-023-03992-y 37462836

[B2] Almeida E. R. Melo A. M. Xavier H. (2000). Toxicological evaluation of the hydro-alcohol extract of the dry leaves of Peumus boldus and boldine in rats. Phytother. Res. 14 (2), 99–102. 10.1002/(sici)1099-1573(200003)14:2<99::aid-ptr600>3.0.co;2-4 10685105

[B3] Álvarez-Abril M. C. García-Alcover I. Colonques-Bellmunt J. Garijo R. Pérez-Alonso M. Artero R. (2023). Natural compound boldine lessens myotonic dystrophy type 1 phenotypes in DM1 Drosophila models, patient-derived cell lines, and HSA(LR) mice. Int. J. Mol. Sci. 24 (12), 9820. 10.3390/ijms24129820 37372969 PMC10298378

[B4] Asencio M. Delaquerrière B. Cassels B. K. Speisky H. Comoy E. Protais P. (1999). Biochemical and behavioral effects of boldine and glaucine on dopamine systems. Pharmacol. Biochem. Behav. 62 (1), 7–13. 10.1016/s0091-3057(98)00096-3 9972839

[B5] Bannach R. Valenzuela A. Cassels B. K. Nunez-Vergara L. J. Speisky H. (1996). Cytoprotective and antioxidant effects of boldine on tert-butyl hydroperoxide-induced damage to isolated hepatocytes. Cell. Biol. Toxicol. 12 (2), 89–100. 10.1007/BF00143359 8738478

[B6] Billaud M. Lohman A. W. Straub A. C. Looft-Wilson R. Johnstone S. R. Araj C. A. (2011). Pannexin1 regulates α1-adrenergic receptor-mediated vasoconstriction. Circ. Res. 109 (1), 80–85. 10.1161/CIRCRESAHA.110.237594 21546608 PMC3135971

[B7] Boeing T. Mariano L. N. B. Dos Santos A. C. Tolentino B. Vargas A. C. de Souza P. (2020). Gastroprotective effect of the alkaloid boldine: involvement of non-protein sulfhydryl groups, prostanoids and reduction on oxidative stress. Chem. Biol. Interact. 327, 109166. 10.1016/j.cbi.2020.109166 32531310

[B8] Bol M. Wang N. De Bock M. Wacquier B. Decrock E. Gadicherla A. (2017). At the cross-point of connexins, calcium, and ATP: blocking hemichannels inhibits vasoconstriction of rat small mesenteric arteries. Cardiovasc Res. 113 (2), 195–206. 10.1093/cvr/cvw215 27677282

[B9] Borisenko S. N. Lekar A. V. Maksimenko E. V. Khizrieva S. S. Borisenko N. I. Minkin V. I. (2020). Synthesis of phenanthrene alkaloids in subcritical water using secoboldine as an example. Chem. Nat. Compd. 56 (1), 183–184. 10.1007/s10600-020-02981-9

[B10] Bourgoin E. Verne D. (1872). Sur l’existence d’un alcali organique dans le bold. Pharm. Chim. (16), 191–193.

[B11] Burrell J. C. Vu P. T. Alcott O. J. B. Toro C. A. Cardozo C. Cullen D. K. (2023). Orally administered boldine reduces muscle atrophy and promotes neuromuscular recovery in a rodent model of delayed nerve repair. Front. Cell. Neurosci. 17, 1240916. 10.3389/fncel.2023.1240916 37829672 PMC10565860

[B12] Cafferata E. A. Castro-Saavedra S. Fuentes-Barros G. Melgar-Rodríguez S. Rivera F. Carvajal P. (2021). Boldine inhibits the alveolar bone resorption during ligature-induced periodontitis by modulating the Th17/Treg imbalance. J. Periodontol. 92 (1), 123–136. 10.1002/JPER.20-0055 32490537

[B13] Cea L. A. Cisterna B. A. Puebla C. Frank M. Figueroa X. F. Cardozo C. (2013). *De novo* expression of connexin hemichannels in denervated fast skeletal muscles leads to atrophy. Proc. Natl. Acad. Sci. U. S. A. 110 (40), 16229–16234. 10.1073/pnas.1312331110 24043768 PMC3791696

[B14] Cea L. A. Fernández G. Arias-Bravo G. Castillo-Ruiz M. Escamilla R. Brañes M. C. (2020). Blockade of hemichannels normalizes the differentiation fate of myoblasts and features of skeletal muscles from dysferlin-deficient mice. Int. J. Mol. Sci. 21 (17), 6025. 10.3390/ijms21176025 32825681 PMC7503700

[B15] Cea L. A. Vásquez W. Hernández-Salinas R. Vielma A. Z. Castillo-Ruiz M. Velarde V. (2023). Skeletal muscle atrophy induced by diabetes is mediated by non-selective channels and prevented by boldine. Biomolecules 13 (4), 708. 10.3390/biom13040708 37189454 PMC10136059

[B16] Cederbaum A. I. Kukiełka E. Speisky H. (1992). Inhibition of rat liver microsomal lipid peroxidation by boldine. Biochem. Pharmacol. 44 (9), 1765–1772. 10.1016/0006-2952(92)90070-y 1333206

[B17] Cermanova J. Kadova Z. Zagorova M. Hroch M. Tomsik P. Nachtigal P. (2015). Boldine enhances bile production in rats via osmotic and farnesoid X receptor dependent mechanisms. Toxicol. Appl. Pharmacol. 285 (1), 12–22. 10.1016/j.taap.2015.03.004 25771127

[B18] Cermanova J. Prasnicka A. Dolezelova E. Rozkydalova L. Hroch M. Chládek J. (2016). Pharmacokinetics of boldine in control and Mrp2-deficient rats. Physiol. Res. 65 (Suppl. 4), S489–S497. 10.33549/physiolres.933520 28006931

[B19] Chandan P. Dev A. Ezhilarasan D. Shree Harini K. (2023). Boldine treatment induces cytotoxicity in human colorectal carcinoma and osteosarcoma cells. Cureus 15 (11), e48126. 10.7759/cureus.48126 38046745 PMC10693387

[B20] Chiang J. Y. L. Ferrell J. M. (2020). Bile acid receptors FXR and TGR5 signaling in fatty liver diseases and therapy. Am. J. Physiol. Gastrointest. Liver Physiol. 318 (3), G554–G573. 10.1152/ajpgi.00223.2019 31984784 PMC7099488

[B21] Chuliá S. Moreau J. Naline E. Noguera M. A. Ivorra M. D. D'Ocón M. P. (1996). The effect of S-(+)-boldine on the alpha 1-adrenoceptor of the Guinea-pig aorta. Br. J. Pharmacol. 119 (7), 1305–1312. 10.1111/j.1476-5381.1996.tb16039.x 8968536 PMC1915823

[B22] Cisterna B. A. Vargas A. A. Puebla C. Fernández P. Escamilla R. Lagos C. F. (2020). Active acetylcholine receptors prevent the atrophy of skeletal muscles and favor reinnervation. Nat. Commun. 11 (1), 1073. 10.1038/s41467-019-14063-8 32103010 PMC7044284

[B23] de Lima N. M. Ferreira E. d. O. Fernandes M. Y. S. D. Lima F. A. V. Neves K. R. T. do Carmo M. R. S. (2017). Neuroinflammatory response to experimental stroke is inhibited by boldine. Behav. Pharmacol. 28, 223–237. 10.1097/FBP.0000000000000265 27763892

[B24] Ezhilarasan D. Raghunandhakumar S. (2021). Boldine treatment protects acetaminophen-induced liver inflammation and acute hepatic necrosis in mice. J. Biochem. Mol. Toxicol. 35 (4), e22697. 10.1002/jbt.22697 33393705

[B25] Fernández G. Arias-Bravo G. Bevilacqua J. A. Castillo-Ruiz M. Caviedes P. Sáez J. C. (2020). Myofibers deficient in connexins 43 and 45 expression protect mice from skeletal muscle and systemic dysfunction promoted by a dysferlin mutation. Biochim. Biophys. Acta Mol. Basis Dis. 1866 (8), 165800. 10.1016/j.bbadis.2020.165800 32305450

[B26] Garré J. M. Retamal M. A. Cassina P. Barbeito L. Bukauskas F. F. Sáez J. C. (2010). FGF-1 induces ATP release from spinal astrocytes in culture and opens pannexin and connexin hemichannels. Proc. Natl. Acad. Sci. U. S. A. 107 (52), 22659–22664. 10.1073/pnas.1013793107 21148774 PMC3012468

[B27] Gerhardt D. Bertola G. Dietrich F. Figueiró F. Zanotto-Filho A. Moreira Fonseca J. C. (2014). Boldine induces cell cycle arrest and apoptosis in T24 human bladder cancer cell line via regulation of ERK, AKT, and GSK-3β. Urol. Oncol. 32 (1), 36.e1–e9. 10.1016/j.urolonc.2013.02.012 24239461

[B28] Gómez G. I. Fernández P. Velarde V. Sáez J. C. (2018). Angiotensin II-induced mesangial cell damaged is preceded by cell membrane permeabilization due to upregulation of non-selective channels. Int. J. Mol. Sci. 19 (4), 957. 10.3390/ijms19040957 29570626 PMC5979336

[B29] Gómez G. I. Velarde V. (2018). Boldine improves kidney damage in the goldblatt 2K1C model avoiding the increase in TGF-β. Int. J. Mol. Sci. 19 (7), 1864. 10.3390/ijms19071864 29941815 PMC6073111

[B30] Gotteland M. Jimenez I. Brunser O. Guzman L. Romero S. Cassels B. K. (1997). Protective effect of boldine in experimental colitis. Planta Med. 63 (4), 311–315. 10.1055/s-2006-957689 9270374

[B31] Graham Z. A. Siedlik J. A. Toro C. A. Harlow L. Cardozo C. P. (2023). Boldine alters serum lipidomic signatures after acute spinal cord transection in male mice. Int. J. Environ. Res. Public Health 20 (16), 6591. 10.3390/ijerph20166591 37623175 PMC10454893

[B32] Guo A. Zhang H. Li H. Chiu A. García-Rodríguez C. Lagos C. F. (2022). Inhibition of connexin hemichannels alleviates neuroinflammation and hyperexcitability in temporal lobe epilepsy. Proc. Natl. Acad. Sci. U. S. A. 119 (45), e2213162119. 10.1073/pnas.2213162119 36322757 PMC9659366

[B33] Heidari R. Moezi L. Asadi B. Ommati M. M. Azarpira N. (2017). Hepatoprotective effect of boldine in a bile duct ligated rat model of cholestasis/cirrhosis. PharmaNutrition 5 (3), 109–117. 10.1016/j.phanu.2017.07.001

[B34] Hernandez-Salinas R. Vielma A. Z. Arismendi M. N. Boric M. P. Sáez J. C. Velarde V. (2013). Boldine prevents renal alterations in diabetic rats. J. Diabetes Res. 2013, 593672. 10.1155/2013/593672 24416726 PMC3876708

[B35] Huang C. Han X. Li X. Lam E. Peng W. Lou N. (2012). Critical role of connexin 43 in secondary expansion of traumatic spinal cord injury. J. Neurosci. 32 (10), 3333–3338. 10.1523/JNEUROSCI.1216-11.2012 22399755 PMC3569730

[B36] Ivorra M. D. Chuliá S. Lugnier C. D'Ocon M. P. (1993). Selective action of two aporphines at alpha 1-adrenoceptors and potential-operated Ca2+ channels. Eur. J. Pharmacol. 231 (2), 165–174. 10.1016/0014-2999(93)90445-n 8384112

[B37] Jimenez I. Speisky H. (2000). Biological disposition of boldine: *in vitro* and *in vivo* studies. Phytother. Res. 14 (4), 254–260. 10.1002/1099-1573(200006)14:4<254::aid-ptr582>3.0.co;2-m 10861968

[B38] Kajiwara Y. Wang E. Wang M. Sin W. C. Brennand K. J. Schadt E. (2018). GJA1 (connexin43) is a key regulator of Alzheimer’s disease pathogenesis. Acta Neuropathol. Commun. 6 (1), 144. 10.1186/s40478-018-0642-x 30577786 PMC6303945

[B39] Kang J. J. Cheng Y. W. (1998). Effects of boldine on mouse diaphragm and sarcoplasmic reticulum vesicles isolated from skeletal muscle. Planta Med. 64 (1), 18–21. 10.1055/s-2006-957358 9491763

[B40] Kang J. J. Cheng Y. W. Fu W. M. (1998). Studies on neuromuscular blockade by boldine in the mouse phrenic nerve-diaphragm. Jpn. J. Pharmacol. 76 (2), 207–212. 10.1254/jjp.76.207 9541284

[B41] Katafuchi T. Makishima M. (2022). Molecular basis of bile acid-FXR-FGF15/19 signaling Axis. Int. J. Mol. Sci. 23 (11), 6046. 10.3390/ijms23116046 35682726 PMC9181207

[B42] Kringstein P. Cederbaum A. I. (1995). Boldine prevents human liver microsomal lipid peroxidation and inactivation of cytochrome P4502E1. Free Radic. Biol. Med. 18 (3), 559–563. 10.1016/0891-5849(94)e0138-9 9101247

[B43] Lau Y. S. Tian X. Y. Huang Y. Murugan D. Achike F. I. Mustafa M. R. (2013a). Boldine protects endothelial function in hyperglycemia-induced oxidative stress through an antioxidant mechanism. Biochem. Pharmacol. 85 (3), 367–375. 10.1016/j.bcp.2012.11.010 23178655

[B44] Lau Y. S. Tian X. Y. Mustafa M. R. Murugan D. Liu J. Zhang Y. (2013b). Boldine improves endothelial function in diabetic db/db mice through inhibition of angiotensin II-mediated BMP4-oxidative stress cascade. Br. J. Pharmacol. 170 (6), 1190–1198. 10.1111/bph.12350 23992296 PMC3838694

[B45] Leboeuf M. Cavé A. Provost J. Tiberghien R. Forgacs P. (1980). Alkaloids of Monimia rotundifolia pet.-th.; preparation of laurotetanine derived compounds with antiarrhythmic properties (author's transl). Ann. Pharm. Fr. 38 (6), 537–544. 7283352

[B46] Lee S.-S. Chiou C. M. Lin H. Y. Chen C. H. (1995). Preparation of phenanthrene alkaloids via solvolysis of 2-hydroxyaporphines. Tetrahedron Lett. 36 (9), 1531–1532. 10.1016/0040-4039(95)00077-p

[B47] Lillo M. A. Muñoz M. Rhana P. Gaul-Muller K. Quan J. Shirokova N. (2023). Remodeled connexin 43 hemichannels alter cardiac excitability and promote arrhythmias. J. Gen. Physiol. 155 (7), e202213150. 10.1085/jgp.202213150 37191672 PMC10192603

[B48] Moezi L. Yahosseini S. Jamshizadeh A. Pirsalami F. (2019). Acute boldine treatment induces anti-convulsant effects in mice through its antioxidant activity. Drug Res. (Stuttg) 69 (4), 227–233. 10.1055/a-0659-2478 30081409

[B49] Mollataghi A. Coudiere E. Hadi A. H. A. Mukhtar M. R. Awang K. Litaudon M. (2012). Anti-acetylcholinesterase, anti-α-glucosidase, anti-leishmanial and anti-fungal activities of chemical constituents of Beilschmiedia species. Fitoterapia 83 (2), 298–302. 10.1016/j.fitote.2011.11.009 22119096

[B50] Moreno P. R. Vargas V. M. Andrade H. H. Henriques A. T. Henriques J. A. (1991). Genotoxicity of the boldine aporphine alkaloid in prokaryotic and eukaryotic organisms. Mutat. Res. 260 (2), 145–152. 10.1016/0165-1218(91)90002-4 2046695

[B51] Namballa H. K. Madapa S. Sigalapalli D. K. Harding W. W. (2022). Semisynthetic transformations on (+)-Boldine reveal a 5-HT(2A/2C)R antagonist. J. Nat. Prod. 85 (9), 2149–2158. 10.1021/acs.jnatprod.2c00365 36001775

[B52] O'Brien P. Carrasco-Pozo C. Speisky H. (2006). Boldine and its antioxidant or health-promoting properties. Chem. Biol. Interact. 159 (1), 1–17. 10.1016/j.cbi.2005.09.002 16221469

[B53] O'Carroll S. J. Gorrie C. A. Velamoor S. Green C. R. Nicholson L. F. B. (2013). Connexin43 mimetic peptide is neuroprotective and improves function following spinal cord injury. Neurosci. Res. 75 (3), 256–267. 10.1016/j.neures.2013.01.004 23403365

[B54] Oliveira Sa A. Pimentel T. Oliveira N. (2020). Boldo-induced hepatotoxicity: a case of unexplained jaundice. Eur. J. Case Rep. Intern Med. 7 (12), 002116. 10.12890/2020_002116 33457373 PMC7806290

[B55] Pandurangan A. K. Mohebali N. Hasanpourghadi M. Looi C. Y. Mustafa M. R. Mohd Esa N. (2016). Boldine suppresses dextran sulfate sodium-induced mouse experimental colitis: NF-κB and IL-6/STAT3 as potential targets. Biofactors 42 (3), 247–258. 10.1002/biof.1267 26891685

[B56] Panzitt K. Zollner G. Marschall H. U. Wagner M. (2022). Recent advances on FXR-targeting therapeutics. Mol. Cell. Endocrinol. 552, 111678. 10.1016/j.mce.2022.111678 35605722

[B57] Paydar M. Kamalidehghan B. Wong Y. L. Wong W. F. Looi C. Y. Mustafa M. R. (2014). Evaluation of cytotoxic and chemotherapeutic properties of boldine in breast cancer using *in vitro* and *in vivo* models. Drug Des. Devel Ther. 8, 719–733. 10.2147/DDDT.S58178 PMC4057328 24944509

[B58] Piscaglia F. Leoni S. Venturi A. Graziella F. Donati G. Bolondi L. (2005). Caution in the use of boldo in herbal laxatives: a case of hepatotoxicity. Scand. J. Gastroenterol. 40 (2), 236–239. 10.1080/00365520410009537 15764158

[B59] Potter L. A. Toro C. A. Harlow L. Lavin K. M. Cardozo C. P. Wende A. R. (2023). Assessing the impact of boldine on the gastrocnemius using multiomics profiling at 7 and 28 days post-complete spinal cord injury in young male mice. Physiol. Genomics 55, 297–313. 10.1152/physiolgenomics.00129.2022 37125768 PMC10292965

[B60] Qiu X. Shi L. Zhuang H. Zhang H. Wang J. Wang L. (2017). Cerebrovascular protective effect of boldine against neural apoptosis via inhibition of mitochondrial bax translocation and cytochrome C release. Med. Sci. Monit. 23, 4109–4116. 10.12659/msm.903040 28841638 PMC5584841

[B61] Retamal M. A. Cortés C. J. Reuss L. Bennett M. V. L. Sáez J. C. (2006). S-nitrosylation and permeation through connexin 43 hemichannels in astrocytes: induction by oxidant stress and reversal by reducing agents. Proc. Natl. Acad. Sci. U. S. A. 103 (12), 4475–4480. 10.1073/pnas.0511118103 16537412 PMC1450196

[B62] Retamal M. A. Schalper K. A. Shoji K. F. Bennett M. V. L. Sáez J. C. (2007). Opening of connexin 43 hemichannels is increased by lowering intracellular redox potential. Proc. Natl. Acad. Sci. U. S. A. 104 (20), 8322–8327. 10.1073/pnas.0702456104 17494739 PMC1895948

[B63] Ribarič S. (2012). The pharmacological properties and therapeutic use of apomorphine. Molecules 17 (5), 5289–5309. 10.3390/molecules17055289 22565480 PMC6268166

[B64] Ribeiro R. J. Silvestre C. Duarte C. (2017). Hidden risks of alternative medicines: a case of boldo-induced hepatotoxicity. J. Diet. Suppl. 14 (2), 186–190. 10.1080/19390211.2016.1207123 27576017

[B65] Sáez J. C. Contreras-Duarte S. Gómez G. I. Labra V. C. Santibañez C. A. Gajardo-Gómez R. (2018). Connexin 43 hemichannel activity promoted by pro-inflammatory cytokines and high glucose alters endothelial cell function. Front. Immunol. 9, 1899. 10.3389/fimmu.2018.01899 30158937 PMC6104120

[B66] Sana-Ur-Rehman H. Markus I. Moore K. H. Mansfield K. J. Liu L. (2017). Expression and localization of pannexin-1 and CALHM1 in porcine bladder and their involvement in modulating ATP release. Am. J. Physiol. Regul. Integr. Comp. Physiol. 312 (5), R763–R772. 10.1152/ajpregu.00039.2016 28254749

[B67] Silva L. C. L. de Souza G. H. Pateis V. d. O. Ames-Sibin A. P. Silva B. P. Bracht L. (2023). Inhibition of gluconeogenesis by boldine in the perfused liver: therapeutical implication for glycemic control. Int. J. Hepatol. 2023, 1283716. 10.1155/2023/1283716 37056327 PMC10089784

[B68] Speisky H. Cassels B. K. (1994). Boldo and boldine: an emerging case of natural drug development. Pharmacol. Res. 29 (1), 1–12. 10.1016/1043-6618(94)80093-6 8202440

[B69] Speisky H. Cassels B. K. Lissi E. A. Videla L. A. (1991a). Antioxidant properties of the alkaloid boldine in systems undergoing lipid peroxidation and enzyme inactivation. Biochem. Pharmacol. 41 (11), 1575–1581. 10.1016/0006-2952(91)90156-y 2043147

[B70] Speisky H. Squella J. A. Núñez-Vergara L. J. (1991b). Activity of boldine on rat ileum. Planta Med. 57 (6), 519–522. 10.1055/s-2006-960197 1818341

[B71] Subramaniam N. Kannan P. K A. Thiruvengadam D. (2019). Hepatoprotective effect of boldine against diethylnitrosamine-induced hepatocarcinogenesis in wistar rats. J. Biochem. Mol. Toxicol. 33 (12), e22404. 10.1002/jbt.22404 31593341

[B72] Tavares D. C. Takahashi C. S. (1994). Evaluation of the genotoxic potential of the alkaloid boldine in mammalian cell systems *in vitro* and *in vivo* . Mutat. Res. 321 (3), 139–145. 10.1016/0165-1218(94)90038-8 7513064

[B73] Toledo J. P. Fernández-Pérez E. J. Ferreira I. L. Marinho D. Riffo-Lepe N. O. Pineda-Cuevas B. N. (2021). Boldine attenuates synaptic failure and mitochondrial deregulation in cellular models of alzheimer's disease. Front. Neurosci. 15, 617821. 10.3389/fnins.2021.617821 33679301 PMC7933475

[B74] Tomšík P. Mičuda S. Muthná D. Čermáková E. Havelek R. Rudolf E. (2016). Boldine inhibits mouse mammary carcinoma *in vivo* and human MCF-7 breast cancer cells *in vitro* . Planta Med. 82 (16), 1416–1424. 10.1055/s-0042-113611 27611982

[B75] Toro C. A. Johnson K. Hansen J. Siddiq M. M. Vásquez W. Zhao W. (2023). Boldine modulates glial transcription and functional recovery in a murine model of contusion spinal cord injury. Front. Cell. Neurosci. 17, 1163436. 10.3389/fncel.2023.1163436 37416508 PMC10321410

[B76] Urzúa A. (1983). Alkaloids from the bark of Peumus boldus. Fitoterapia 54 (4), 175–177.

[B77] Walstab J. Wohlfarth C. Hovius R. Schmitteckert S. Röth R. Lasitschka F. (2014). Natural compounds boldine and menthol are antagonists of human 5-HT3 receptors: implications for treating gastrointestinal disorders. Neurogastroenterol. Motil. 26 (6), 810–820. 10.1111/nmo.12334 24708203

[B78] Yi C. Ezan P. Fernández P. Schmitt J. Sáez J. C. Giaume C. (2017). Inhibition of glial hemichannels by boldine treatment reduces neuronal suffering in a murine model of Alzheimer's disease. Glia 65 (10), 1607–1625. 10.1002/glia.23182 28703353

[B79] Zagorova M. Prasnicka A. Kadova Z. Dolezelova E. Kazdova L. Cermanova J. (2015). Boldine attenuates cholestasis associated with nonalcoholic fatty liver disease in hereditary hypertriglyceridemic rats fed by high-sucrose diet. Physiol. Res. 64 (Suppl. 4), S467–S476. 10.33549/physiolres.933206 26681076

[B80] Zetler G. (1988). Neuroleptic-like, anticonvulsant and antinociceptive effects of aporphine alkaloids: bulbocapnine, corytuberine, boldine and glaucine. Arch. Int. Pharmacodyn. Ther. 296, 255–281. 2907279

[B81] Zhang C. Yan Z. Maknojia A. Riquelme M. A. Gu S. Booher G. (2021). Inhibition of astrocyte hemichannel improves recovery from spinal cord injury. JCI Insight 6 (5), e134611. 10.1172/jci.insight.134611 33682795 PMC8021110

[B82] Zhao H. Xu H. Qiao S. Lu C. Wang G. Liu M. (2017). Boldine isolated from Litsea cubeba inhibits bone resorption by suppressing the osteoclast differentiation in collagen-induced arthritis. Int. Immunopharmacol. 51, 114–123. 10.1016/j.intimp.2017.08.013 28826044

